# Meiosis in rare males in parthenogenetic *Cacopsylla myrtilli* (Wagner, 1947) (Hemiptera, Psyllidae) populations from northern Europe

**DOI:** 10.3897/CompCytogen.v7i3.6126

**Published:** 2013-09-30

**Authors:** Christina Nokkala, Valentina G. Kuznetsova, Seppo Nokkala

**Affiliations:** 1 Laboratory of Genetics, Department of Biology, University of Turku, FI-20014, Turku Finland; 2 Department of Karyosystematics, Zoological Institute, Russian Academy of Sciences, St.Petersburg 199034, Russia

**Keywords:** *Cacopsylla myrtilli*, parthenogenesis, spanandric males, asynaptic meiosis, nonfunctional males

## Abstract

For studying meiosis in males, large samples of *Cacopsylla myrtilli* (Wagner, 1947) (Hemiptera, Psyllidae) were collected in Norway, Sweden, Finland and northwest Russia. In addition to all-female populations, males were present in 10 out of 47 populations; still, all populations were highly female-biased, the proportion of males varying from 0.1% to 9.1%. These males are thus rare or so-called spanandric males. Males in northern Norway, Finland and northwest Russia showed normal chiasmate meiosis, while complete absence of chiasmata due to asynapsis was found in males collected in Norway and northern Sweden. In asynaptic meiosis, all univalent chromosomes divided during the first meiotic division resulting in incomplete second meiotic division and formation of diploid sperms. Hence, males in these populations are nonfunctional and do not contribute to the genetic constitution of the population, but appear in every generation as reversals from apomictic parthenogenesis and the mode of parthenogenesis is of obligatory type.

## Introduction

For bisexual reproduction it is essential that the diploid chromosome complement is reduced to haploid during gametogenesis. This is achieved by a specialized cell cycle, meiosis, where the chromosome number of germ line cells is reduced during two rounds of cell divisions after just one round of chromosome replication. In the first, reductional division homologous chromosomes segregate from each other while in the other, equational division individual chromosomes divide. During early prophase of the first meiotic division, homologous chromosomes pair, undergo tight synapsis and form a bivalent. Bivalent integrity is retained throughout prophase with either of two ways. Most often a crossing-over occurs during synapsis creating a physical link or chiasma between the homologs, which are held together until anaphase I. Alternatively, in achiasmate meiosis, pairing, alignment and synapsis are normal, but no chiasmata are formed. In these cases, the paired condition of homologs is retained till the onset of anaphase I (for references see Nokkala and Nokkala 1983, Nokkala 1987, Grozeva et al. 2010).

In natural populations, the meiotic mechanism is under strong selection; all mutations causing disturbances in meiosis are quickly removed from the population. The only exceptions are thelytokous parthenogenetic populations, where males are occasionally found (Lynch 1984, Palmer and Norton 1990). In these rare males defects affecting normal meiotic pattern and making males nonfunctional in sexual reproduction have been described. In two oribatid mite species *Trhrypochthonius tectorum* (Berlese, 1896) and *Platynothrus peltifer* (CL Koch, 1839) (Acari: Oribatida: Desmonomata) male meiosis was normal, but spermiogenesis was aberrant; males produced few spermatophores only and females left them without attention (Taberly 1988). In the thelytokous false spider mite *Brevipalpus obovatus* Donnadieu, 1875 (Phytoptipalpidae) males had low sperm production and did not inseminate females despite copulation (Pinjacker et al. 1981). Spermatogonia are developed into sperm without meiosis in the spider mite *Tetranychus urticae* Koch, 1836 (Tetranychidae) (Pijnacker and Drenth-Diephuis 1972).

The object of our study is the Holarctic psyllid species *Cacopsylla myrtilli* (Wagner, 1947), which has long been considered as parthenogenetic (Linnavuori 1951, Lauterer 1963, Ossiannilsson 1975, 1992), since collections have been made from all-female populations (Ossiannilsson 1992). Recently, we established that parthenogenetic females in *Cacopsylla myrtilli* were triploids and displayed parthenogenesis of an apomictic type (Nokkala et al. 2008). However, also males have been found in some populations in northwest Russia (Ossianilsson 1975, Labina et al. 2009), Norway (Hodkinson 1983), northern Sweden (Hodkinson and Bird 2006) and northern Finland (Labina et al. 2009). Based on the presence of males, either facultative parthenogenesis (Hodkinson 1983, Hodkinson and Bird 2006) or even bisexual reproduction (Labina et al. 2009) has been suggested.

For the present study we have collected *Cacopsylla myrtilli* from many populations from Norway including recollection of Hodkinson (1983), Sweden including recollection of Hodkinson and Bird (2006), and Finland. For northwest Russia, we have utilized the collection of Labina et al. (2009). We have analyzed populations quantitatively for the presence of males and when present studied male meiosis in detail to reveal possible aberrations and their significance for the meiotic mechanism in general. In addition we wanted to find firm evidence for the origin of males in populations.

## Materials and methods

Adult *Cacopsylla myrtilli* specimens were collected in Norway, Sweden, Finland and northwest Russia. For the present study, we selected only those populations, where males were found (Table 1). The Rindhovda population in Norway has been earlier studied by Hodkinson (1983) and the Abisko population in Sweden by Hodkinson and Bird (2006).

Specimens were fixed immediately after collection in the field in freshly made 3:1 Carnoy and stored in fixative in the laboratory at + 6°C until slides were made according to the method by Nokkala et al. (2008). For males collected in alcohol in the field, a novel approach was used. The abdomen of a male was immersed in fresh 3:1 Carnoy fixative for two hours, testes were dissected in a drop of 45% acetic acid and squashed. This procedure allows both chromosomal and molecular analysis of the same individual.

**Table 1.** Locations of *Cacopsylla myrtilli* populations in Norway, Sweden, Finland and Russia. Number of cytologically studied males, total number of males, number of females and frequency of males.

**Population**	**latitude, longitude**		**altitude**	**males**		**females**	**male freq.**
				**studied**	**total**		
**Norway**							
Finnmark, Šuoššjávri	69°22'11"N, 24°18'20"E			3	4	2950	0.001
Sjoa, Rudihøe	61°46'27"N, 9°17'16"E		1000	1	3	1078	0.003
Sjoa, Kvernbrusætrin	61°42'27"N, 9°19'25"E		950	15	22	1443	0.015
Sjoa, Stålane	61°41'15"N, 9°14'27"E		1000	11	14	470	0.030
Sjoa, Kringlothaugen	61°43'06"N, 9°22'40"E		700	1	1	277	0.004
Sjoa, Rindhovda	61°43'05"N, 9°05'12"E		1080	30	38	379	0.091
**Sweden**							
Abisko, Lapporten	68°19'26"N, 18°51'05"E		610	5	5	386	0.013
**Finland**							
Utsjoki	69°51'06"N, 27°00'34"E			2	3	2603	0.001
Paltamo	64°33'28"N, 27°43'41"E			6	10	1595	0.006
**Russia**							
White Sea, Sredny Island^†^	66°17'00"N, 33°40'00"E			9	49	2946	0.016

^†^Data from Labina et al. (2009)

For staining of chromosomes the Feulgen-Giemsa method by Grozeva and Nokkala (1996) was employed with extending staining in Schiff’s reagent to 25 min and subsequent Giemsa staining in Sørensen’s buffer, pH 6.8, to 30 min.

Chromosomes were photographed with BU4-500C CCD camera (BestScope International Limited, Beijing, China) mounted on Olympus BX51 microscope (Olympus, Japan) using ISCapture Sofware version 2.6 (Xintu Photonics Co LTD, Xintu, China). Photographs were processed with Corel Photo-Paint X5 software.

## Results

We found males in ten (Table 1) out of a total of 47 populations collected. All populations studied were highly female-biased, male frequency varying from 0.1 % to 9.1 %. The highest proportion of males was found from Rinhovda population in Norway collected above tree line. This same population was earlier studied by Hodkinson (1983). All other populations showed a considerably lower proportion of males, less than 3 %. The lowest proportion of males or 0.1 % was found in Finnmark, Šuoššjávri in Norway and Utsjoki in Finland.

The testes in male psyllids are organized into lobed structures, the number of which varies in a species specific manner (Głowacka et al. 1995). In *Cacopsylla myrtilli*, males have two lobed testes. The earliest meiotic stage found in an adult male is pachytene, while earlier meiotic stages are rare. The number of cells at pachytene in one primary spermatocyte cyst is 64.

Males collected in Finnmark, Šuoššjávri in Norway, in Paltamo and Utsjoki in Finland and the White Sea area in Russia (Labina et al. 2009), on one hand, and in Sjoa in Norway and in Abisko in northern Sweden, on the other hand, showed strikingly different course of meiosis.

**Figure 1.**
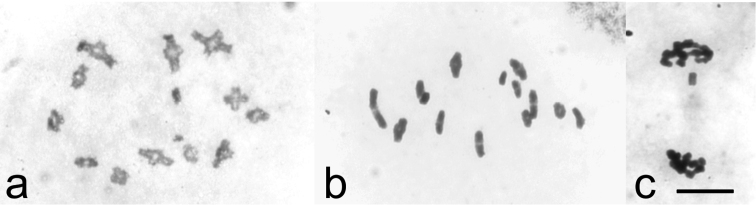
Chiasmate male meiosis in *Cacopsylla myrtilli* (**a**) diakinesis with twelve autosomal bivalents an univalent X chromosome (**b**) metaphase I with twelve chiasmate bivalents and univalent X (**c**) telophase I, univalent X chromosome moving towards upper pole. Bar equals 10 µm.

Males from northern Norway, Finland and Russia showed meiotic stages typical for psyllids known to possess holocentric chromosomes. The most common stage in testes was the diffuse stage during which chromatin had a diffuse appearance covering the whole nucleus in a cell. During this stage the size of cells is increased considerably. When the chromosomes condensed out from the diffuse stage, diplotene stage with 12 autosomal bivalents with one chiasma in each and a univalent X chromosome were seen (Fig. 1a). At metaphase I, the bivalents showed axial orientation with homologous telomeres oriented to opposite poles and the univalent X chromosome lying at the equatorial plane (Fig. 1b). Segregation of bivalents at anaphase I resulted in half-bivalents moving towards the poles. The bi-oriented X chromosome was seen as a laggard at this stage. At telophase I, the X chromosome moved to one of the poles (Fig. 1c), resulting in two kinds of secondary spermatocytes, those with the X chromosome and those without it.

**Figure 2.**
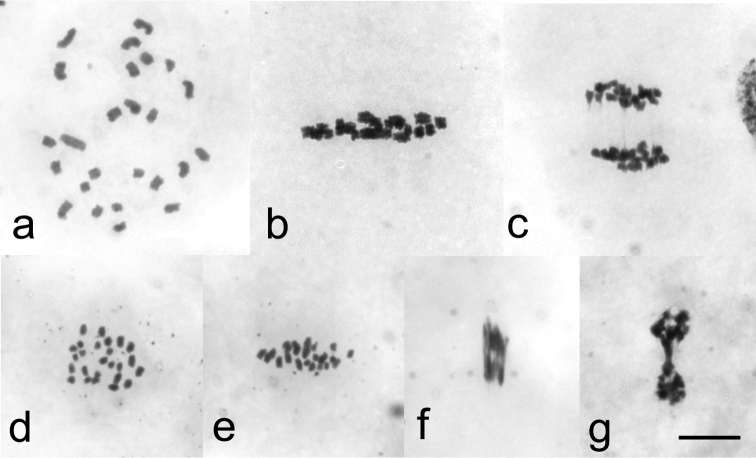
Male meiosis without chiasmata in *Cacopsylla myrtilli* (**a**) Diakinesis with 25 univalent chromosomes (**b**) Metaphase I in side view with 25 bi-oriented univalents (**c**) Anaphase I, daughter chromosomes moving towards opposite poles. No laggard chromosomes present (**d**) Metaphase II in polar view showing 25 daughter chromosomes (**e**) Metaphase II in side view. All bi-oriented daughter chromosomes aligned with the equatorial plane (**f**) Anaphase II, daughter chromosomes stretched towards poles (**g**) Telophase II, daughter nuclei joined by stretched chromosomes. Bar equals 10 µm.

However, males collected near Sjoa, Norway (Rindhovda, Rudihøe, Kvernbrusætrin and Stålane) and northern Sweden (Abisko, Lapporten) showed 25 (24 + X) univalent chromosomes at diplotene and diakinesis stages, indicating a complete failure in chiasma formation (Fig. 2a). Univalent chromosomes oriented with sister chromatids to opposite poles at metaphase I (Fig. 2b). These bi-oriented chromosomes divided at anaphase I. No laggard was seen at anaphase I, indicating that the X chromosome also divided in the first meiotic division (Fig. 2c). Anaphase I resulted in secondary spermatocytes with 25 daughter chromosomes, oriented to both poles at metaphase II (Fig. 2d, e). As daughter chromosomes are unable to divide, anaphase II started but could not be completed (Fig. 2f, g). Consequently, diploid spermatids were produced. Meiosis resulted in a cyst of 128 developing spermatids instead of the 256 in normal chiasmate meiosis.

**Figure 3.**
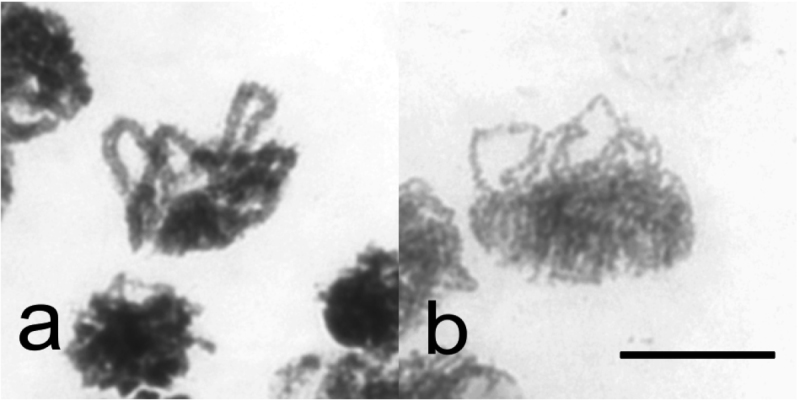
Synapsis and synaptic alignment of chromosomes in *Cacopsylla myrtilli* (**a**) Pachytene, synapsed chromosomes in bouquet orientation in chiasmate male meiosis (**b**) Presynaptic alignment of leptotene chromosomes in male meiosis lacking chiasmata in bouquet orientation. Bar equals 10 µm.

To find out reasons for the absence of chiasmata, early stages of meiosis were analysed in more detail. In chiasmate male meiosis pachytene stage (Fig. 3a) was easily found in all males studied. However, in males without chiasmata no pachytene cells were found in 61 males studied, but leptotene-like nuclei were abundant. When seen in side-view chromosomes showed distinct bouquet orientation (Fig. 3b), indicating that presynaptic alignment was normal, but synapsis did not occur. It seems apparent then that the failure in chiasma formation in these males is due to a mutation resulting in asynapsis of homologous chromosomes.

## Discussion

### Males in populations

In the present study we have established that none of the male-carrying populations in Norway, northern Sweden, Finland and northwest Russia were genuinely bisexual, but all were parthenogenetic with highly female-biased sex ratio. Variation of male frequencies from 0.1 to 9.1 % was quite similar to that previously found in parthenogenetic oribatid mites, in which frequencies are varying from 0.15 % to 6.3 % (Palmer and Norton 1990). On the other hand, in bisexual species of mites, the proportion of males is over 30 % (Maraun et al. 2003). Clearly, males in *Cacopsylla myrtilli* populations represent rare or so-called spanandric males. Surprisingly, two kinds of males were found. Meiosis in males was normal in northern populations in Norway, Finland and Russia. Generally it is thought that rare males are nonfunctional (Lynch 1984, Palmer and Norton 1990, Maraun et al. 2003, Smith et al. 2006). However, the significance of rare males in these *Cacopsylla myrtilli* populations is difficult to evaluate at present, as we do not know, if there are diploid females with normal meiosis within triploid parthnogenetic populations and if the males are able to make a distinction between triploid and diploid females. These problems are at present under investigation. The low frequencies of males in the populations, 0.1% in Utsjoki, 0.6% in Paltamo and 1.6% in White Sea might indicate that they are not capable of independent bisexual reproduction in these populations. In the remaining populations, due to asynapsis, only univalents were present in meiosis resulting in the formation of diploid spermatids. Evidently, males in these populations are nonfunctional, not being able to produce diploid offspring with any kind of females and not contributing thus to the genetic constitution of the population. Apparently, males appear in every generation as reversals from apomictic parthenogenesis and reproduction in the populations is of obligatory parthenogenetic type.

### Univalents and their meiotic behavior

Although complete failure in chiasma formation in male meiosis was found for the first time in a natural population in the present study, the phenomenon is well known in the holocentric laboratory model organism, the nematode *Caenorhabditis elegans* (Maups, 1900). Genetic dissection studies on early events in meiosis, presynaptic alignment, formation of double strand breaks, synapsis and crossing over, and chiasma formation, have revealed several gene loci, in which mutations result in complete lack of crossing over in an affected animal (for reviews see Garcia-Muse and Boulton 2007, Zetka 2009). Consequently, mutants show twelve univalents in diakinesis stage nuclei instead of six bivalents described in wild type male. Univalent chromosomes behave in two ways during meiotic divisions. In *spo-11* mutant, univalent chromosomes are unable to divide in the first meiotic division and distribute randomly to poles at first anaphase, resulting in highly aneuploid gametes. In mutants *rec8, htp1, htp2* and *htp3*, univalent chromosomes undergo equational first division. The daughter chromosomes cannot divide in the second division and are all included in the gametes formed. Consequently, if for example *rec8* mutants are mated with normal wild type animals, triploid offspring will be produced (Severson et al. 2009). It is obvious that univalent chromosomes in *Cacopsylla myrtilli* male meiosis in Rindhovda population behave similarly as the univalents in the *rec8* and *htp* mutants in *Caenorhabditis elegans*.

### Cell cycle checkpoints are not activated despite of extensive asynapsis

Both mitosis and meiosis include surveillance mechanisms to ensure regular behavior of chromosomes. In meiosis, homologous chromosomes pair and align with each other followed by their tight synapsis that allows exchange process or crossing over between homologs necessary for maintaining bivalent configuration later in meiotic prophase. These early stages are controlled by the pachytene checkpoint. Defects in synapsis or recombination result in meiotic arrest or apoptosis, thus preventing the formation of defective gametes (Li et al. 2009). The checkpoint is very robust in mammalian spermatogenesis, for example in male mouse asynapsis results in apoptosis or meiotic arrest (Hamer et al. 2008, Kouznetsova et al. 2011), but is less effective in female mouse (Kouznetsova et al. 2007). There are some indications that asynapsis activates apoptosis in the nematode *Caenorhabditis elegans* (Bhalla and Dernburg 2005), but still the behavior of univalents in meiotic mutants in this species seems to be regular in meiotic divisions (Severson et al. 2009). As found in the present study, the pachytene checkpoint is totally inactive in *Cacopsylla myrtilli* male meiosis despite extensive asynapsis. It appears to be inactive also in other insects. In *Drosophila melanogaster* Meigen, 1830, univalents resulted from an asynaptic mutation *c*(*3*)*G* behave in a regular way and reach anaphase I-like stage in mature eggs in female meiosis (Puro and Nokkala 1977). Univalents reach MI also in lepidopteran species (Nayak 1978) and plants (Couteau et al. 1999).

The orientation of chromosomes in metaphase spindle is monitored by the spindle assembly checkpoint. As far as even one chromosome does not reach a proper spindle microtubule attachment to the spindle pole, the onset of anaphase in mitosis or anaphase I or anaphase II in meiosis is inhibited. Once all the chromosomes display proper orientation, the onset of anaphase is triggered by anaphase promoting complex that activates separase enzyme that will degrade sister chromatid cohesion (Nasmyth 2001, Musacchio and Salmon 2007). In *Cacopsylla myrtilli*, univalents behave in a regular way both in the first and second divisions in male meiosis without any disturbances. Apparently, stable bipolar orientations of univalents at MI and daughter chromosomes at MII allow them to evade the spindle assembly checkpoint. This observation is well in accordance with the behavior of univalents in mouse (Kouznetsova et al. 2007).

### Segregation of univalent chromosomes in meiosis

In meiosis, there are mechanisms, which are responsible for the regular segregation of univalent chromosomes, especially in the case of specialized chromosomes like sex chromosomes (Nokkala 1986a), m-chromosomes (Nokkala 1986b) or B-chromosomes (Nokkala 1986c, Nokkala and Nokkala 2004). Typical for these mechanisms is that they can ensure the regular segregation of only one pair of univalents. If more than one pair of univalents are present, regular segregation is disturbed (Nokkala 1986c). Asynaptic meiosis in *Cacopsylla myrtilli* male clearly indicates that if all chromosomes appear as univalents in meiosis there is no mechanism to ensure their segregation but asynapsis leads to highly uneuploid gametes or diploid gametes depending on the behavior of univalents in the first meiotic division. *Drosophila* female meiosis is an exception as asynaptic mutants like *c*(*3*)*G* can be maintained as homozygote mutant stocks, since segregation of homologous chromosomes is still highly regular. Regular segregation is ensured by distributive segregation mechanism operating in the female (Puro and Nokkala 1977, Hawley and Theurkauf 1993).

## Conclusions

In this study we have established that males occurred in ten out of 47 *Cacopsylla myrtilli* populations sampled in northern Europe. All populations were highly female-biased, the proportion of males varying from 0.1 % to 9.1 %. Thus, all populations are in fact parthenogenetic and males are rare or so-called spanandric males. In northern Norway, Finland and Russia male meiosis was normal whereas in Norway and Sweden males displayed asynaptic meiosis leading to the formation of diploid spermatids. These males are nonfunctional in reproduction and appear in every generation as reversals from apomictic parthenogenesis.
